# Psychometric Properties of the Short Form of the Fear of Cancer Recurrence Inventory (FCRI) in Chinese Breast Cancer Survivors

**DOI:** 10.3389/fpsyt.2019.00537

**Published:** 2019-08-07

**Authors:** Li Peng, Weirong Huang, Wenmo Zhang, Yuanyuan Xu, Fang Lu, Ling Zhong, Xianchun Chen, Song Xu, Wenjun Chen, Min Li

**Affiliations:** ^1^Department of Military Psychology, School of Psychology, Third Military Medical University, Chongqing, China; ^2^Xiamen Cancer Hospital, The First Affiliated Hospital of Xiamen University, Xiamen, China; ^3^Department of Fundamental, Army Logistical University of PLA, Chongqing, China; ^4^Breast Center of Southwest Hospital, Third Military Medical University, Chongqing, China; ^5^Psychiatry Department, No.991 Hospital of Chinese Liberation Army, Xiangyang, China

**Keywords:** cancer, oncology, fear of cancer recurrence, anxiety, depression, breast cancer survivors

## Abstract

**Objective:** Currently, fear of cancer recurrence (FCR) is emerging as an important issue for long-term breast cancer survivors and is associated with lower quality of life and functional impairment. Given that there is a dearth of research regarding the FCR of Chinese breast cancer survivors, this study investigated whether the short form of the Fear of Cancer Recurrence Inventory (FCRI) could detect high FCR and explored the level and characteristics of FCR in breast cancer survivors.

**Methods:** Two hundred forty patients who had undergone successful breast cancer surgery in China submitted their survey through a website. The participants’ demographic and medical data, level of FCR, anxiety, depression, and quality of life were assessed.

**Results:** Two hundred seven patients with ages ranging from 19 to 60 years completed the questionnaires. The mean FCR score of the total sample was 18.39. A cutoff score of 12 or higher on the short form of the FCRI was optimal for the detection of high FCR with a sensitivity of 98.6% and a specificity of 35%, and the PPV (positive predictive values) and NPV (negative predictive values) were 44% and 98%, respectively. The area under the curve of the receiver operating characteristics (ROC) analysis was 83%. A total of 159 breast cancer survivors (76.81%) experienced high FCR levels (FCR score > 12), characterized by lower functional and overall health than survivors with a low FCR (*P* < 0.01).

**Conclusions:** The short form of the FCRI is capable of detecting high FCR and is therefore able to assist Chinese breast cancer survivors in receiving appropriate care for reducing FCR.

## Background

Breast cancer is the most common cancer among women worldwide (1). In China, breast cancer has been the leading cause of cancer incidence in women (2). According to the GLOBOCAN statistics, the incidence of breast cancer is up to 21.6/10,000 in Chinese females (3). Rapid advancements in cancer treatments, early cancer diagnosis, and better treatment strategies have led to continuous increases in survival rates (4), but breast cancer and its treatment are often accompanied by physical and psychological impairment and reduction in quality of life (5). With more attention paid to the life-after-treatment phase, many breast cancer survivors experience multiple physical and psychosocial long-term and delayed effects of treatment (e.g., fatigue, pain, and anxiety) (6–8).

Emerging evidence has demonstrated that fear of cancer recurrence (FCR) is a problematic long-term and delayed effect experienced by cancer survivors. Cancer survivors report an unmet need for help dealing with FCR. FCR is defined as worry or anxiety that cancer could recur or develop in the same place or spread to another part of the body (9). Previous researchers report prevalence rates of FCR ranging from 37% to 99% among breast cancer survivors (10–12). Moderate FCR is expected and adaptive (e.g., holding an appropriate vigilance for signs of recurrence, maintaining medical follow-up, and engaging in healthy lifestyle). However, excessive FCR may adversely impact survivors’ emotions and social activities (12, 13). It was found that cancer survivors with high FCR often experience more self-focus and excessive psychological distress (14). In addition, from a public health standpoint, FCR is related to increased health-care utilization and costs (15, 16). In a recently published study, FCR was the most distressful and commonly reported problem by patients with breast cancer (17, 18).

Currently, researchers have developed many tools to assess cancer survivors’ FCR, such as the *Fear of Recurrence Questionnaire* (FRQ) (19), the *Fear of Progression Questionnaire* (FoP-Q) (20), and the *Concerns about Recurrence Scale* (CARS) (21). Among these scales, *the Fear of Cancer Recurrence Inventory* (FCRI) has been verified to possess good specificity and sensitivity and is best suited for diverse cancer populations (22). This 42-item inventory is a multidimensional measure that evaluates seven components of FCR: triggers (nine items), severity (nine items), psychological distress (four items), coping strategies (nine items), functional impairments (six items), insight (three items), and reassurance (three items) (23). The scale was initially validated in 600 cancer patients with different tumor sites, and it was found that all subscales had good internal consistency and reliability, and the 1-month retest reliability ranged from medium to high (24). In China, the Chinese version of the FCRI has been assessed in a sample of 240 cancer survivors; the scale possessed good reliability and validity and could be used to measure fear of cancer recurrence (25). Subsequently, researchers found in clinical application that the severity factor score of the scale was highly correlated with the total score. Therefore, the severity factor of the FCRI (nine items in total) was recommended as a short scale for rapid screening of cancer recurrence fear (25). However, the cutoff values were also different in different populations. A study of 60 French-Canadian cancer survivors showed that the nine-item short scale of fear of cancer recurrence showed good psychometric characteristics, and the study considered that the cutoff had high sensitivity and specificity when divided into 13 points (18). More specifically, a receiver operating characteristic analysis showed that this cutoff score was associated with a sensitivity of 88% and a specificity of 75%. Additionally, Fardell and his colleagues examined 240 Australian cancer survivors using the short scale of fear of cancer recurrence and found that 22 was the optimal cutoff score for screening high and low cancer recurrence fear (26). However, the capacity of the short scale to screen patients for FCR and identify the presence of clinically significant levels of FCR among a large sample of Chinese breast cancer survivors has not yet been demonstrated.

There is a lack of a gold standard measure for FCR and no unified definition of what constitutes a clinical level of FCR. Previous studies showed that a higher FCR was generally associated with increased anxiety in cancer survivors. It is possible to use the subscale of the Hospital Depression and Anxiety Scale as the gold standard measure of the short form of the FCRI for the evaluation of FCR. Determining the cutoff point using the short form of the FCRI is beneficial for screening high-risk patients, and these patients could receive interventions to lower their fear of cancer recurrence.

Previous studies have revealed that FCR has detrimental impacts on cancer survivors’ quality of life. Weert and colleagues found that approximately 30% of survivors reported decreased quality of life due to physical concerns after their diagnosis and treatment (27). There is evidence that higher levels of FCR are associated with poorer quality of life among all types of cancer survivors, such as breast, colorectal, lung, pancreatic and periampullary, urogynecologic, and testicular cancer survivors (28). However, until recently, few studies have explored the characteristics related to Chinese breast cancer survivors’ FCR and the differences in quality of life between patients with low FCR and high FCR based on the cutoff point.

Therefore, the aims of this study were a) to assess the capacity of the short form of the Fear of Cancer Recurrence Inventory to screen the clinical levels of FCR in Chinese breast cancer survivors and b) to compare the quality of life between breast cancer survivors with high FCR and low FCR.

## Methods

### Participants

This study utilized a cross-sectional observational research design. Patients were recruited from a website over a recruitment period of 3 months (from May to July 2017). The inclusion criteria were as follows: a) confirmed first diagnosis of stage 0–III breast cancer; b) age greater than or equal to 18 years; c) no recurrence or metastases; d) completed treatment (i.e., lumpectomy, mastectomy, and radiation but not hormone therapy); and e) able to read and understand Chinese. The exclusion criteria were diagnoses of stage IV cancer, severe cognitive impairments, and psychiatric disorders. A total of 240 breast cancer survivors submitted the survey. Among those, 33 participants were excluded because 21 were currently receiving treatment and 12 experienced cancer recurrence. Finally, 207 participants were eligible for this study.

### Procedure

Ethical approval was obtained from the Ethics Committee of Third Military Medical University of China prior to the start of the study (ref. no. ChiCTR-OOC-17012132). The survey was uploaded to https://www.wjx.cn/jq/13794312.aspx, a website that allows surveys to be taken confidentially online. The researchers posted this survey to a patient network group. These participants logged into the website and read the purpose of the study, and then they needed to complete a booklet containing questionnaires about both demographic and medical information. By returning the booklet, the participants gave their written informed consent to take part in the study.

### Measures

Demographic and medical information was assessed with questions regarding age, marital status, education level, employment, family yearly income, years since diagnosis, cancer stage, and cancer treatments received. With the use of Brislin’s two-way translation model, the translation of all the English versions of the scales was carried out independently by two psychology professionals (29). Then, a psychology expert modified the two translated versions and reached a consensus with the two translators.

#### Fear of Recurrence

The nine-item Fear of Cancer Recurrence Inventory is a short form of the FCRI (nine-item FCRI) and corresponds to the severity subscale of the FCRI (42 items). The nine-item FCRI evaluates the presence and severity of intrusive thoughts associated with FCR. Each item is rated on a Likert scale ranging from 0 (“not at all” or “never”) to 4 (“a great deal’ or “all the time”). A higher score indicates higher levels of FCR. In this study, the reliability coefficient of the Chinese version of the short form of the FCRI was 0.912 (30).

#### Depression and Anxiety

The Hospital Anxiety and Depression Scale (HADS) includes 14 items divided into two subscales (depression and anxiety), each with seven items. Scores obtained for each subscale range from 0 to 21. The more anxiety, depression, and psychological distress the patients suffer from, the higher the scores are. The HADS includes no somatic items that could be confused by signs related to physical illness. In this study, we utilized the scores of the anxiety subscale as the gold standard to differentiate high and low fear of cancer recurrence. A total score of 8 or higher indicated high distress (31). The reliability coefficient of the anxiety subscale was 0.732, and that of the depression subscale was 0.734.

#### Quality of Life

The European Organization for the Research and Treatment of Cancer Quality of Life Questionnaire C30 (EORTC-QLQ-C30) is widely used to assess the quality of life of cancer patients. The scale comprises 30 items covering five function subscales (physical, role, emotional, cognitive, and social), nine common cancer symptoms (fatigue, nausea/vomiting, pain, dyspnea, insomnia, appetite loss, constipation, diarrhea, and financial difficulties), and an overall health status (32). Respondents rate the frequency of occurrence of the former 28 items from 1 (not at all) to 4 (often) and the 29th and 30th items from 1 (worst) to 7 (best). The higher the scores in the functional domain and overall quality of life, the better the health status and quality of life. The higher the score in the symptom domain, the worse the quality of life. Symptoms were not examined in this study. Cronbach’s alpha coefficient of the scale is 0.962.

### Data Analyses

Before the data were analyzed using SPSS (version 18.0), the normality of the relevant data was verified, and the distribution was found to be normal. Patients were categorized into two groups according to their anxiety scores: patients with clinical levels of anxiety (i.e., anxiety score ≥ 8) and patients with nonclinical levels of anxiety (i.e., anxiety score < 8). Receiver operating characteristics (ROC) analysis was conducted to evaluate the performance of the short form of the FCRI. The accuracy properties of sensitivity, specificity, and positive and negative predictive values were assessed at each cutoff point of the short form of the FCRI. Furthermore, the area under the ROC curve and its 95% confidence interval were examined. To differentiate high FCR from low FCR, an optimal cutoff point should have high sensitivity and specificity, which maximizes the proportion of patients whose test results are accurate. Independent *t* tests were performed for anxiety and depression to assess differences between individuals with high and low FCR based on the cutoff score.

## Results

### Participant Characteristics

The age of the participants ranged from 19 to 60 years. Approximately 47.34% of the participants belonged to the 40–50-year age group. The majority of the participants (86.96%) were married. In terms of education level, 29.47% of the subjects had a tertiary education, while 7.73% had a primary education. Most participants were unemployed (39.13%), and 54.59% of them earned less than 50,000 RMB ($7,908) as their family yearly income. In terms of the clinical characteristics of the participants, 81.65% of the participants reported that it was less than 5 years since their breast cancer diagnosis. The sociodemographic characteristics and clinical features of the participants are presented in **Table 1**.

**Table 1:** Demographic and medical characteristics of the sample.

Descriptive characteristics	*n* (%)	Descriptive characteristics	*n* (%)
Age (years)		Family income per year (RMB)	
Age < 30	2 (0.96%)	Below RMB 50,000	113 (54.59%)
30 ≤ Age < 40	36 (17.39%)	RMB 50,000 to 100,000	74 (35.75%)
40 ≤ Age < 50	98 (47.34%)	RMB 100,000 to 300,000	15 (7.25%)
Age ≥ 50	71 (34.31%)	More than RMB 300,000	5 (2.41%)
**Marital status**		**Years since cancer diagnosis**	
Single	3 (1.45%)	Time ≤ 2 years	83 (40.10%)
Married	180 (86.96%)	2 years < Time ≤ 5 years	86 (41.55%)
Divorced	18 (8.69%)	5 Years < Time ≤ 10 years	30 (14.49%)
Widowed	6 (2.90%)	More than 10 years	8 (3.86%)
**Education level**		**Cancer stage**	
Primary school	16 (7.73%)	I	50 (24.15%)
Secondary school	63 (30.43%)	II	127 (61.35%)
Diploma/professional	67 (32.37%)	III	30 (14.50%)
University degree	61 (29.47%)	IV	0 (0%)
**Employment**		**Cancer treatments received**	
Full time	55 (26.57%)	Mastectomy	148 (71.50%)
Unemployed	81 (39.13%)	Conservative therapy	28 (13.53%)
Retired	71 (34.30%)	Mastectomy + breast construction	31 (14.97%)

### ROC Analysis

**Figure 1:**
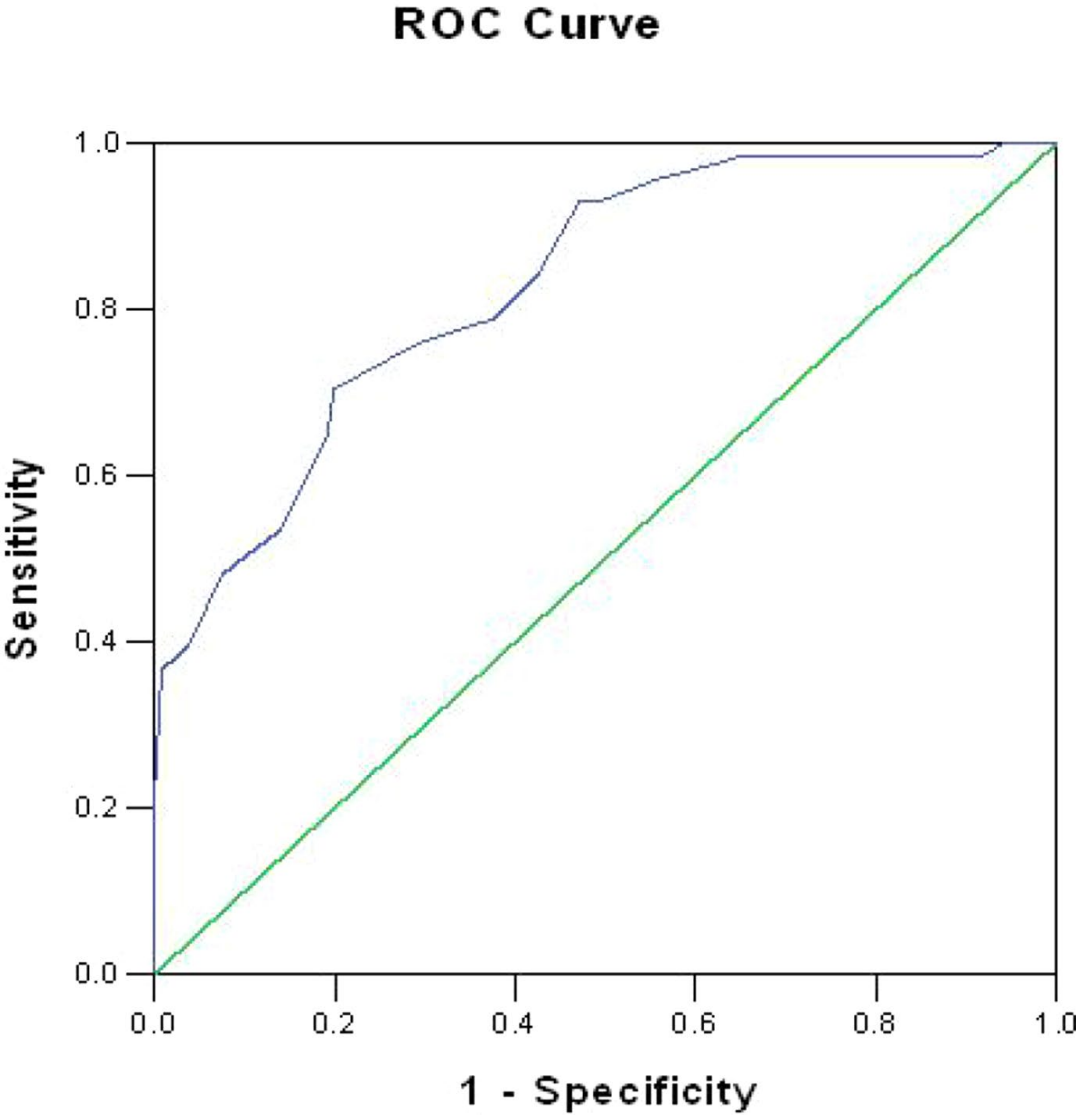
Receiver operating characteristics (ROC) curve for the short form of the Fear of Cancer Recurrence Inventory.

The area under the curve of the ROC analysis was 83% (*P* < 0.001; 95% CI = 0.773–0.887), suggesting a good level of diagnostic accuracy (see **Figure 1**). **Table 2** presents the sensitivity and specificity rates obtained for a sample of scores from the short form of the FCRI. According to our goal to validate a rapid screening tool of FCR, more emphasis was placed on the capacity of the instrument to detect the largest possible number of survivors with clinical levels of FCR (i.e., sensitivity) and to maximize the proportion of negative test results corresponding to nonclinical FCR survivors (i.e., NPV) (30). Based on these criteria, a score of 12 or higher appeared to constitute the optimal clinical cutoff score to differentiate between high FCR and low FCR, with a sensitivity of 98.6% and a specificity of 35%. The PPV (positive predictive values) and NPV (negative predictive values) were 44% and 98%, respectively.

**Table 2:** Performance of the short form of the Fear of Cancer Recurrence Inventory (FCRI) as a screening tool to detect clinical FCR (*n* = 207).

Cutoff score	Sensitivity (%)	Specificity (%)	1 − Specificity (%)	PPV (%)	NPV (%)
7	100.0	5.8	94.2	35.5	100.0
8	98.6	8.8	91.2	35.9	92.3
9	98.6	16.1	83.9	37.8	95.7
10	98.6	27.0	73.0	41.2	97.4
11	98.6	32.8	67.2	43.2	97.8
**12**	**98.6**	**35.0**	**65.0**	**44.0**	**98.0**
13	97.2	40.1	59.9	45.7	96.5
14	95.8	46.0	54.0	47.9	95.5
15	93.0	54.0	46.0	51.2	93.7
16	93.0	55.4	44.6	51.6	93.9
17	84.5	65.0	35.0	55.6	89.0
18	78.9	73.0	27.0	60.2	87.0
19	76.1	82.5	17.5	69.2	86.9
20	63.1	74.5	25.5	60.7	76.4
21	56.9	83.7	16.3	68.5	75.7
22	52.3	85.6	14.4	69.4	74.2
23	43.8	89.4	10.6	72.2	71.8
24	40.0	92.8	7.2	77.6	71.2
25	37.7	94.2	5.8	80.3	70.8
26	30.8	96.6	3.4	85.1	69.1

PPV, positive predictive value; NPV, negative predictive value.

12 or higher = optimal cutoff to screen for high FCR.

### Comparisons of Quality of Life Between Breast Cancer Survivors With High FCR and Low FCR

The mean score of FCR was 18.39 (7.14). Among the 207 breast cancer survivors, 159 (76.81%) were considered to display high FCR levels (FCR score > 12), while 48 patients (23.19%) showed low fear of cancer recurrence. **Table 3** demonstrates that breast cancer survivors with high FCR have significantly lower functional and overall health than have survivors with low fear (*P* < 0.01).

**Table 3:** Differences in quality of life between survivors with high fear of cancer recurrence (FCR) and low FCR.

	High fear (*n* = 159)	Low fear (*n* = 48)	*t*
	M (SD)	M (SD)	
Physical functioning	68.44 (26.62)	82.86 (15.46)	−4.54**
Role functioning	74.17 (25.06)	84.27 (18.63)	−2.92**
Emotional functioning	68.89 (28.59)	80.78 (13.65)	−3.83**
Cognitive functioning	67.22 (25.18)	78.74 (14.37)	−3.87**
Social functioning	71.67 (27.49)	81.80 (17.01)	−2.98**
Overall health	42.76 (22.24)	67.62 (16.54)	−6.92**

** P < 0.01.

## Discussion

The aims of our study were to assess the capacity of the short form of the FCRI to screen for clinical levels of FCR among Chinese breast cancer survivors and to compare the quality of life between breast cancer survivors with high FCR and those with low FCR. Our results demonstrated that a cutoff score of 12 or higher on the short form of the FCRI had optimal sensitivity and specificity values for screening clinical FCR. Additionally, our results revealed that breast cancer survivors with high FCR were significantly more likely to have lower quality of life than cancer survivors with low FCR.

Studies have explored the cutoff value of cancer survivors’ clinical levels of FCR using an FCR questionnaire in many countries. Simard and Savard found that the short form of FCRI was a rapid and effective tool to screen clinical levels of FCR, and a cutoff score of 13 or higher on the FCRI-SF was found in 60 French-Canadian mixed cancer survivors (21). Custers and his colleagues explored whether the Cancer Worry Scale (CWS) could be used as a tool to identify high levels of fear of recurrence in breast cancer survivors in the Netherlands, and the results suggested that a score of 14 or higher on the CWS was optimal for detecting severe levels of FCR (33). Similarly, they found that a cutoff score greater than or equal to 14 on the CWS was optimal for the detection of high FCR in colorectal cancer survivors (34). However, to our knowledge, few studies have explored the screening capacity of the FCRI and a cutoff point among Chinese breast cancer survivors. As already mentioned, obtaining a cutoff point is quite challenging given the absence of a “gold standard” measure and definition of FCR. Our study revealed a cutoff score of 12 or higher on the short form of the FCRI, which was lower than the cutoff scores in other countries. A possible reason for this difference is that our study was conducted with only females and breast cancer survivors. Additionally, Chinese breast cancer survivors may lack cancer knowledge and may be more likely to misunderstand the association between cancer and death. Moreover, many cancer survivors have negative beliefs that cancer is incurable, which may increase their FCR (35–37).

This study revealed that FCR is very prevalent in Chinese breast cancer survivors. In accordance with previous studies, cancer survivors with a high level of FCR reported lower functional and overall health. To our knowledge, once diagnosed, breast cancer survivors may experience psychological distress to some degree, which could negatively affect quality of life and well-being. Many studies have found that increased FCR was related to emotional distress and poor mental health in cancer survivors, which has a detrimental effect on their quality of life, including physical and psychological functioning (38, 39). These findings suggest that FCR is a common experience among cancer survivors and that some survivors report more fear than do others. Hence, cancer survivors with high levels of FCR need to receive better psychological treatment.

## Study Limitations

This study has several possible limitations. First, although the sample size can meet the requirements of the study, there may still be some selection bias. Therefore, it is necessary to further expand the sample size for testing because the relatively small sample size of our study may reduce the power of between-group comparisons and would limit the generalization of the prevalence rates of FCR among Chinese breast cancer survivors. In the future, our findings need to be replicated with a larger sample of Chinese breast cancer survivors. Second, the cutoff score is limited by the absence of gold standard criteria to diagnose FCR, and the specificity of our study was very low because our goal was to validate a rapid screening tool for FCR to detect the largest possible number of survivors with clinical levels of FCR (i.e., sensitivity) and to maximize the proportion of negative test results corresponding to nonclinical FCR survivors (i.e., NPV). Additionally, the cross-sectional design of our study does not make it possible to determine whether anxiety and depression were risk factors or consequences of FCR.

## Clinical Implications

The study provides empirical support for the short form of the FCRI to be used as an optimal tool to detect high FCR and determined a cutoff point. This finding enables researchers to screen breast cancer survivors with high FCR and could guide doctors to pay attention to patients’ FCR. Future research needs to develop psychological interventions that decrease FCR and improve quality of life among Chinese breast cancer survivors.

## Conclusion

In summary, the short form of the FCRI is able to detect high levels of FCR among breast cancer survivors. The high prevalence of FCR indicated that particular attention should be paid to Chinese females with breast cancer. Early psycho-education and management focusing on FCR specifically need to be provided for breast cancer survivors.

## Ethics Statement

Ethical approval was obtained from the Ethics Committee of Third Military Medical University of China prior to the start of the study (ref. no.: ChiCTR-OOC-17012132).

## Author Contributions

YX, FL, LZ, XC, SX and WC collected the data. WH, WZ and LP analyzed the data, LP and ML designed the experiment and wrote this paper.

## Funding

This work was supported by the National Natural Science Fund (No. 31700958) and Chongqing Technology Innovation and Application Demonstration Project (No. cstc2018jscx-msybX0119).

## Conflict of Interest Statement

The authors declare that the research was conducted in the absence of any commercial or financial relationships that could be construed as a potential conflict of interest.
